# Food and Nutrient Intake among 12-Month-Old Norwegian-Somali and Norwegian-Iraqi Infants

**DOI:** 10.3390/nu8100602

**Published:** 2016-09-28

**Authors:** Navnit Kaur Grewal, Lene Frost Andersen, Cathrine Solheim Kolve, Ingrid Kverndalen, Liv Elin Torheim

**Affiliations:** 1 Department of Nursing and Health Promotion, Faculty of Health Sciences, Oslo and Akershus University College of Applied Sciences, P.O. Box 4 St. Olavs Plass, 0130 Oslo, Norway; cathrinekolve@gmail.com (C.S.K.); ingrid.kverndalen@hotmail.com (I.K.); liv.elin.torheim@hioa.no (L.E.T.); 2 Department of Nutrition, Institute of Basic Medical Sciences, University of Oslo, P.O. Box 1046 Blindern, 0317 Oslo, Norway; l.f.andersen@medisin.uio.no

**Keywords:** food, nutrient intake, infants, immigrants, Norway

## Abstract

The aim of the present paper was to describe food and nutrient intake among 12-month-old Norwegian-Somali and Norwegian-Iraqi infants, with a focus on iron and vitamin D intake. A cross-sectional survey was conducted from August 2013 through September 2014. Eighty-nine mothers/infants of Somali origin and 77 mothers/infants of Iraqi origin residing in Eastern Norway participated in the study. Data were collected using two 24-h multiple-pass recalls. Forty percent of the Norwegian-Somali infants and 47% of the Norwegian-Iraqi infants were breastfed at 12 months of age (*p* = 0.414). Median energy percentages (E%) from protein, fat and carbohydrates were within the recommended intake ranges, except the level of saturated fats (12–13 E%). Median intakes of almost all micronutrients were above the recommended daily intakes. Most of the infants consumed iron-enriched products (81%) and received vitamin D supplements (84%). The median intakes of iron and vitamin D were significantly higher among infants receiving iron-enriched products and vitamin D supplements compared to infants not receiving such products (*p* < 0.001). The findings indicate that the food and nutrient intake of this group of infants in general seems to be in accordance with Norwegian dietary recommendations. Foods rich in iron and vitamin D supplements were important sources of the infants’ intake of iron and vitamin D and should continue to be promoted.

## 1. Introduction

The first two years of a child’s life are particularly important as this is a period of rapid growth when nutritional needs are high [1,2]. Knowledge about infants’ and toddlers’ food and nutrient intake is therefore important to evaluate their diet and identify potential challenges. In Norway, national dietary surveys of infants and toddlers have been performed at the ages of 6, 12 and 24 months [3,4,5]. Due to methodological issues, infants and toddlers of immigrant mothers were excluded from these surveys, and separate surveys for these children have been called for by the Norwegian Directorate of Health [6].

Consequently, compared to infants of Norwegian-born parents, less is known about the food and nutrient intake of infants of immigrant parents in Norway. However, some previous smaller studies of Norwegian immigrant children have shown some potential nutrition challenges, such as high prevalence of iron and vitamin D deficiencies and a relatively high added sugar intake [7,8,9]. It has further been suggested that immigrant children may be introduced to cow’s milk at a younger age than ethnic Norwegian children, although inconsistent trends have been observed [9]. As cow’s milk contains very little iron, consumption >500 mL/day has been associated with reduced iron stores in young children [10,11]. However, intake of fortified foods seems to improve iron status in late infancy [12].

To address the paucity of data on food and nutrient intake and infant feeding practices among Norwegian children with immigrant backgrounds, the InnBaKost study was initiated in 2012. Information on breastfeeding and complementary feeding practices among 6-month-old Norwegian-Somali and Norwegian-Iraqi infants was recently published [13]. The present paper aimed to describe and compare food and nutrient intake among 12-month-old Norwegian-Somali and Norwegian-Iraqi infants, with a focus on iron and vitamin D intake.

## 2. Materials and Methods

### 2.1. Subjects and Design

A cross-sectional survey was carried out from August 2013−September 2014. Mothers born in Somalia or Iraq and living in Oslo, Akershus or Buskerud were eligible for inclusion. These two immigrant groups were chosen, as they are the two non-Western immigrant groups in Norway with the highest number of births per year [14]. Children on a special diet due to serious illness were excluded. If the mother had twins or triplets, only one child was included by random selection.

Mothers who participated in the InnBaKost survey of 6-month-old infants [13] were contacted again when their children turned 12 months old. These mothers had not received any information about the results in the 6-months survey or dietary counselling which could influence dietary habits at the infants’ 12 months of age. In the 6-month survey, 107 Norwegian-Somali mothers and infants and 80 Norwegian-Iraqi mothers and infants participated [13]. Of these, 70 Norwegian-Somali infants and 61 Norwegian-Iraqi infants participated in the 12-month follow-up survey. Seven of the Somali-born mothers and eight of the Iraqi-born mothers declined to participate further, while 30 Somali-born mothers and 11 Iraqi-born mothers could not be reached. Along with the mothers/infants participating in the 6-month survey, 19 additional Somali-born mothers and 16 additional Iraqi-born mothers were recruited. The additional mothers were recruited through lists obtained from the National Population Register. Mothers who were not reached from these lists in the 6-month survey were approached again when the infants were 12 months. A total sample of 89 Norwegian-Somali infants and 77 Norwegian-Iraqi infants were included for the present analysis.

To collect data on the food intake of the 12-month-old infants, two 24-h multiple-pass recalls were conducted face-to-face as closely as possible to the child’s 12-month birthday. Eight trained female fieldworkers speaking the relevant language performed the interviews in Somali, Arabic, Kurdish or Norwegian. A total of 8% (*n* = 13) only completed one single 24-h recall. For mothers who were unable to meet in person, the interview was conducted over the telephone. Of all the participants, 3% (*n* = 5) had both interviews conducted over the telephone, while 4% (*n* = 7) had one of the two interviews conducted over the telephone. The interviews were conducted at a time and place chosen by individual mothers. The average time between the two 24-h recalls was 2 weeks. For 7% of the participants, there were more than 4 weeks between the two interviews. Approximately 78% of all the dietary recalls were collected for intakes on weekdays and 22% were collected for intakes on weekends among Norwegian-Somali infants, and the numbers were 83% and 17%, respectively, for Norwegian-Iraqi infants. In both groups, over 85% of the recall days were reported as representative of the usual daily dietary intake of the child. Each mother who completed the two 24-h recalls received a voucher for a baby shop worth approximately US $25.

The Regional Committees for Medical and Health Research Ethics (2012/957) approved the study, and informed consent was obtained from all the participating mothers.

### 2.2. 24-h Multiple-Pass Recall Method

The 24-h multiple-pass recall method was used to collect data on the 12-month-old Norwegian-Somali and Norwegian-Iraqi infants. The method was first developed and pilot tested on 12 Somali-born and Iraqi-born mothers from January to June 2013 [15].

Briefly, in this dietary recall method, the mothers were to be interviewed twice about the exact food and beverage intake of their children during the preceding 24 h. A 24-h period was defined as from the time the child woke up the previous day until the time the child woke up the day of the interview.

The dietary recall was divided into three passes. In the first pass, the mothers were asked to report everything that their children had consumed the previous day, including during the night. Each breastfeeding occurrence was also to be reported. In the second pass, mothers were asked to provide additional detailed information about each item of food and drink consumed by the children. This included type of product, brand names, cooking methods, amounts and leftovers. For homemade dishes, the mothers were asked for the recipes, and the list of ingredients was recorded. The third pass was a review of the recall, and the mothers were given the opportunity to provide any additional information regarding the children’s food and drink consumption. During the last pass, the field workers were also instructed to prompt for information about foods and drinks not mentioned that were considered to be easy to forget, such as snacks, fruits, water, juices and supplements, which the field workers read from a list.

Two photographic booklets previously used in Norwegian national dietary surveys, were utilized during the interviews as an aid to estimate portion sizes. The *Spedkost* booklet [4] containing 17 colour photograph series of infant portion sizes of selected foods and drinks was mostly employed. The *Norkost* booklet [16] containing a series of adult portion sizes was mainly used to estimate portion sizes of different kinds of bread.

A picture library containing 405 pictures divided into 19 folders on an iPad was used to help identify the types of foods/drinks/products given to the children. The development of the picture library has been described in more detail elsewhere [15].

### 2.3. Questionnaire and Background Characteristics

A questionnaire with 19 questions on five topics was used to collect additional information about the infants. The questions related to breastfeeding (whether the infant was breastfed, and if so, the frequency of breastfeeding), the age of introduction to various foods and drinks, whether the infant had any allergies/intolerances, the use of dietary supplements and arenas for receiving infant nutrition information.

In Norway, healthcare personnel routinely measure the length and weight of children during regular check-ups at children’s health clinics, including at 12 months of age. Participating mothers were asked to bring their infant’s health cards to the interview in order to record these data. Other background characteristics, such as infants’ gender, mothers’ age, mothers’ age when they immigrated to Norway, number of children, parental education levels and fathers’ origins, were already provided from the 6-month survey. The same questions were asked of newly recruited mothers. All mothers were also asked to provide information on current work status and person/place in charge of day-care for the infants. When possible, other persons in charge of the day-care of the infant during the recall days were consulted on the infants’ dietary intake during those hours.

### 2.4. Food Intake and Nutrient Calculations

Information from the 24-h recalls was manually coded by nutritionists. All foods and beverages consumed by each child were converted into grams of edible portions. For recipes, a modified version of the summing method was applied to calculate the intake of each ingredient [17]. A breast milk intake of 124 mL per feed or an intake of 497 mL for children whose mothers reported to have breastfed their children four times or more was used, as described by Grewal et al. [15]. The daily nutrient intakes (averaged over the two days) were computed using a food database and a dietary calculation system (KBS, version 7.2, database AE-10) developed at the Department of Nutrition, University of Oslo, Oslo, Norway. The food database used is mainly based on the official Norwegian food composition table but is also continuously supplemented with data on new food items and nutrient content. Relevant food products missing from KBS were added, and the nutrient content was based on the nutrient content listed on the products’ nutritional label. Nutrients from dietary supplements and from fortified foods consumed during the recall days were included in the calculated nutrient intake. The calculated average daily nutrient intake from the two recalls was compared to the recommended intake ranges for macronutrients and recommended daily intakes (RI) for micronutrients [18].

### 2.5. Statistical Analysis

Data from the questionnaire and background characteristics were manually entered into the statistical software package IBM SPSS Statistics version 22.0. Data about the food and nutrient intakes estimated from KBS were transferred to SPSS. All data were further processed and analysed in SPSS. Energy intake information acquired during the telephone interviews (*n* = 12) did not differ from those who completed two face-to-face 24-h recalls and were therefore included in the analyses. Mothers who only completed one 24-h recall were also included.

The weight and length of the children were converted into Z-scores for weight-for-age, height-for-age and weight-for-height according to 2006 WHO child growth standards [19] using WHO Anthro (version 3.2.2, January 2011, Geneva, Switzerland) and macros. Cow’s milk and yoghurt were categorized as ‘dairy products’ in the analysis. Infant formula and infant cereals were categorized as ‘iron-enriched products’.

Iron intake was compared between infants receiving and not receiving dairy products and between infants receiving and not receiving iron-enriched products. The intake of dairy products and the intake of iron-enriched products were tested by different background characteristics. The intake of vitamin D was compared between infants receiving and not receiving vitamin D supplements. The intake of vitamin D supplements was tested by different background characteristics. The data did not adhere to a normal distribution, and continuous variables are presented as median and 25th and 75th percentiles (P_25_–P_75_) and were tested using a Mann-Whitney U test. Categorical variables were tested using a Chi-square test.

## 3. Results

Selected characteristics of the infants and their parents are presented in Table 1. The Norwegian-Somali infants had significantly higher Z-scores for height-for-age (1.4 vs. 0.6, *p* = 0.001) but not for weight-for-height (0.7 vs. 0.2, *p* = 0.087) compared to the Norwegian-Iraqi infants. The Somali-born mothers were younger (29 years vs. 32 years, *p* = 0.026), had lived in Norway for a longer period of time (10 years vs. 7 years, *p* = 0.012) and a higher percentage were working at the time of interviewing (27% vs. 13%, *p* = 0.026) compared to the Iraqi-born mothers. A higher proportion of the Iraqi-born mothers had higher education compared to the Somali-born mothers (64% vs. 38%, *p* = 0.001).

### 3.1. Breastfeeding

At 12 months of age, 40% of the Norwegian-Somali infants and 47% of the Norwegian-Iraqi infants were breastfed (*p* = 0.414). The median breastfeeding frequency among those breastfeeding was 4.5 times a day in both groups.

### 3.2. Food Intake

Data from the 24-h recalls on daily intakes of various types of foods and drinks among all infants are presented in Table 2. Percentages of and intake among consumers are presented in Table 3. Norwegian-Somali infants had significantly higher median daily intakes of commercial infant cereals, commercial fruit purees, fish/fish products, yoghurt and cow’s milk compared to the Norwegian-Iraqi infants (Table 2). Norwegian-Iraqi infants, on the other hand, had significantly higher daily intakes of refined flour bread, cake, meat and meat products, eggs, fruits and berries, added sugar, tea and water than Norwegian-Somali infants (Table 2).

Commercial infant cereals and fruit purees, potatoes and fish/fish products were consumed by a significantly higher proportion of Norwegian-Somali infants compared to Norwegian-Iraqi infants, while bread, grain products, cake, meat, eggs, fruit/berries and tea were more commonly consumed by Norwegian-Iraqi infants (Table 3).

### 3.3. Energy and Nutrient Intake

The average energy and nutrient intake from the 24-h recalls are shown in Table 4. The median energy intake among Norwegian-Somali infants was 3791 kJ compared to 4122 kJ among Norwegian-Iraqi infants (*p* = 0.059). The median energy percentages (E%) from protein, fat and carbohydrates were within the recommended intake ranges in both groups; however, the intake of saturated fats was above the recommended upper level of 10 E%. The intake of added sugar, on the other hand, was below 5 E% in both groups.

The main sources of saturated fat intake in the total diet of the Norwegian-Somali infants were cow’s milk (which contributed with 22%), breast milk (22%), commercial infant cereals (9%) and cheese (8%) (data not shown). The main sources of saturated fats among the Norwegian-Iraqi infants were breast milk (23%), meat and meat products (11%), cow’s milk (9%) and cheese (9%). Yoghurt was the main source of the added sugar intake among both the Norwegian-Somali and Norwegian-Iraqi infants (38% vs. 23%).

The median intake of all micronutrients, except iron and niacin in both groups and zinc among Norwegian-Somali infants were above the RI.

### 3.4. Factors Associated with Iron Intake

The main sources of dietary iron were commercial infant cereals (which contributed with 51% among Norwegian-Somali vs. 34% among Norwegian-Iraqi infants), iron supplements (13% vs. 9%), infant formula (10% vs. 19%) and bread and other grain products (10% vs. 8%). During the recall days, 6% of the Norwegian-Somali infants and 8% of the Norwegian-Iraqi infants received supplements containing iron (*p* = 0.574, data not shown). Table 5 shows the percentage of the infants receiving dairy products and iron-enriched products and also median intakes of iron among infants receiving dairy products and iron-enriched products. There were no significant differences between the two groups regarding the proportion who had received dairy products, whether it concerned any dairy products or more than 500 g dairy products per day. A higher proportion of Norwegian-Somali infants had received iron-enriched products compared to Norwegian-Iraqi infants (88% vs. 73%, respectively; *p* = 0.015). Intakes of iron did not differ significantly between infants receiving dairy products compared to infants not receiving dairy products. However, the median intake of iron was significantly higher among infants receiving iron-enriched products (8.1 mg/day) compared to infants not receiving iron-enriched products (3.7 mg/day; *p* < 0.001). Neither intake of dairy products nor intake of iron-enriched products differed significantly between genders, according to mothers’ ages, number of years lived in Norway, education level, current work status or number of children.

### 3.5. Factors Associated with Vitamin D Intake

Vitamin D supplements comprised more than half of the vitamin D intake in both groups (61% among Norwegian-Somali infants vs. 52% among Norwegian-Iraqi infants). Other sources of vitamin D were commercial infant cereals (19% in both groups), infant formula (7% vs. 17%) and fish and fish products (6% vs. 3%) among Norwegian-Somali and Norwegian-Iraqi infants, respectively. During the recall days, a total of 84% of the infants had received vitamin D supplements. Significantly more among the Norwegian-Somali infants compared to the Norwegian-Iraqi infants had received vitamin D supplements during the recall days (94% vs. 71%, respectively; *p* < 0.001) The median (P_25_–P_75_) intake of vitamin D among those receiving vitamin D supplements was 14.5 (10.8–18.1) μg/day compared to 4.1 (1.5–7.6) μg/day among those not receiving vitamin D supplements (*p* < 0.001). The intake of vitamin D supplements did not differ significantly between genders, according to mothers’ ages, number of years lived in Norway, education level, current work status or number of children.

## 4. Discussion

The present paper describes the food and nutrient intake in a group of 12-month-old Norwegian infants of mothers who immigrated to Norway from Somalia and Iraq. The findings indicate that the nutrient intake in this group of infants in general seems to be in accordance with Norwegian dietary recommendations, with the possible exception of certain micronutrients. The intake of added sugar was low, whereas the median intake of saturated fats exceeded the recommendations.

### 4.1. Breastfeeding

In this group of infants with immigrant backgrounds, 40% of the Norwegian-Somali infants and 47% of the Norwegian-Iraqi infants were breastfed at 12 months of age, with a median frequency of 4.5 times a day. This is quite similar to the 46% found to be breastfed in the Norwegian national dietary survey of infants at 12 months of age who had an average breastfeeding frequency of 3.5 times a day [4]. Breastfeeding patterns have been shown to vary between ethnic subgroups in a society, but also to depend on country of residence [20,21,22]. Data from a nationally representative population based cohort study from Ireland [21] and the National Survey of Children’s Health in the US [22] reported higher breastfeeding initiation and longer duration among immigrant mothers compared to the native mothers. However, the breastfeeding prevalence in Ireland and the US were in general lower than what is common in Norway and other Scandinavian countries [23]. This may explain the opposite findings and the greater differences between immigrant and native mothers compared to our findings.

### 4.2. Food Intake

Commercial infant cereals, bread, potatoes and vegetables, fruit and berries, meat/meat products and fish/fish products were the most commonly consumed foods among the Norwegian-Somali and Norwegian-Iraqi infants. These foods were also consumed by a high proportion of infants in the Norwegian national dietary survey [4]. Commercial infant dinners were consumed by a higher proportion of infants in the national dietary survey compared to infants in the present survey [4]. Homemade dinners were also more common than commercial infant dinners at 6 months of age among Norwegian-Somali and Norwegian-Iraqi infants [13]. The lower use of commercial infant dinners among the children with immigrant background may be due, as reported in a study of Somali mothers in the US, to concerns about the lack of freshness of commercially produced baby foods and also the practice of feeding children the same foods as adults [24].

One important consideration when comparing these results is that the Norwegian national dietary survey used a semi-quantitative food frequency questionnaire (FFQ) to assess the infants’ dietary intake at 12 months of age [4], while the present study used repeated 24-h recalls due to limited knowledge about Norwegian-Somali and Norwegian-Iraqi infants’ diets. Thus, any comparison between the two studies should be undertaken with caution because the results from the national dietary survey represent the infants’ intake over a longer period of time.

### 4.3. Energy and Nutrient Intake

The energy intake among both Norwegian-Somali and Norwegian-Iraqi infants was lower than the mean intake of 5100 kJ reported in the national dietary survey, which only reported the energy and nutrient intake of non-breastfed infants [4]. The average daily energy requirement for boys and girls at 12 months of age is 337 kJ/kg and 333 kJ/kg, respectively, according to Nordic nutrition recommendations [25]. Based on the median weights of the infants in the sample, Norwegian-Somali infants would have an average daily energy requirement of 3440 kJ and Norwegian-Iraqi infants would have an average daily energy requirement of 3252 kJ, which is lower than the average energy intake found in this study. However, the quality of the data on weight and height may have some limitations as each child health clinic may differ in terms of accuracy in reporting the measurements and the measurements may have been taken a few weeks before or after the 24-h recalls were performed. In addition, the weights of several infants were not registered, which may also have affected the median weights reported in this study. Furthermore, breast milk amounts were only estimated and this, along with possible misreporting of dietary intakes, may give inaccurate energy intake data.

The median E% of protein, total fat and carbohydrates were all within the recommended intake ranges [18]. Saturated fat was above the recommended 10 E%. However, one of the major sources of saturated fats in the diet was breast milk, which is recommended to give to infants until 12 months of age and beyond [18]. Other main sources of saturated fats was cow’s milk and meat and meat products. Parents could reduce the infants’ intake of saturated fats by changing high-fat dairy products with low-fat products as well as using lean cuts of meat. The intake of added sugar was appreciably lower than the recommended upper level of 10 E%, which is a positive finding. These results are quite similar to those reported in the national dietary survey, where saturated fats accounted for 12 E% and added sugar only accounted for 4 E%. Previous studies of young children with immigrant backgrounds in Norway and other countries have suggested that high sugar and sweetened beverage intake could be prevalent in this group [9,26,27,28]. However, the studies from Norway were conducted several years ago, and it could be that the trend of reducing the intake of added sugar, which has been reflected in the national dietary surveys, is also the case for this group of children. In the national dietary surveys, the intake of added sugar decreased from 10 E% in 1999 to 4 E% in 2007 due to the reduced intake of sugary drinks among Norwegian infants and the reduced levels of added sugar in commercial infant products [4,29]. In addition, a qualitative survey among Somali-born mothers in Norway revealed that most mothers thought that their infants at 12 months were too young to be given sugar [30]. However, the older siblings were usually eating candy and the majority of mothers had sweets available at home at all times, which may indicate that sweets and sugary drinks may be introduced at a later stage in the child’s life.

### 4.4. Factors Associated with Iron Intake

Iron deficiency has been reported to be prevalent among both immigrant and non-immigrant children in Europe [7,31,32], however, some studies have suggested a higher prevalence among immigrant children [7,32]. The consumption of milk with low iron content and the excessive consumption of cow’s milk have been reported in the literature to be factors that may cause iron deficiency [32,33]. On the other hand, iron-enriched products may be a protective factor against inadequate iron intake [32].

In the present study, we found no significant differences in iron intake among infants who consumed dairy products compared to those who did not consume dairy products. There were no significant differences among those who consumed more than 500 g/day of dairy products compared to those who consumed less. Still, high consumption of cow’s milk is not recommended as it may inhibit the iron absorption [34]. Higher iron intakes were observed among infants who received iron-enriched products compared to those who did not receive these products. This has also been found among one-year-old children in other studies [12,35]. The majority of infants in the present study received iron-enriched products; however, the median intake of iron was lower than the RI [18] and the intake reported in the national dietary survey (12.8 mg/day) [4] for both Norwegian-Somali and Norwegian-Iraqi infants. The higher intakes of iron among the infants in the national dietary survey may be explained by a lower consumption of cow’s milk (92 g/day), with only 51% consumers, and a higher consumption of commercial infant cereals (293 g/day) and infant formula (144 g/day), with 82% and 40% consumers, respectively [4]. Commercial infant cereals and infant formula were the two major sources of iron in the diet of the infants in the national dietary survey, followed by bread and meat/meat products [4].

### 4.5. Factors Associated with Vitamin D Intake

The intake of vitamin D exceeded the recommended levels for both Norwegian-Somali and Norwegian-Iraqi infants. Severe vitamin D deficiency has been reported to be prevalent among 6-week-old infants of immigrant backgrounds in Norway who did not receive vitamin D supplements [8]. In the present study, vitamin D supplements were given to the majority of Norwegian-Somali and Norwegian-Iraqi infants, as was seen in the 6-month survey [13]. The proportion was lower among the Norwegian-Iraqi infants. Vitamin D supplements were more commonly given in this sample compared to the 67% reported in the national dietary survey; those infants also had lower average intake of vitamin D (6.8 μg/day) [4]. Those not receiving vitamin D supplements in the present survey were more likely to have much lower intakes of vitamin D than those receiving vitamin D supplements and also lower intakes than the RI of 10 μg/day [18]. Vitamin D supplementation has recently been reported by Moffat et al. [36] to be almost similar and high among both immigrants and Canadian-born mothers, although less so for refugee mothers. According to their findings, current public education on vitamin D supplementation delivered by nurses and other health educators seems to be similarly effective for Canadian and immigrant parents [36]. A study by Madar et al. showed significant improvement in vitamin D status among infants with immigrant backgrounds in Norway after receiving free vitamin D supply together with tailor-made information handouts [37]. Vitamin D supplements are provided for free to of non-Western immigrant backgrounds at child health centres in Norway due to concerns about inadequate vitamin D intakes in this group. [38]. The findings of this study suggest that this information may have reached the majority of the participating mothers.

### 4.6. Strengths and Limitations of the Study

Two very specific subgroups of the Norwegian population were chosen for the study, which is an important strength of the survey. According to lists from the National Population Register, the participating Norwegian-Somali and Norwegian-Iraqi infants accounted for approximately 19% and 27%, respectively, of all Norwegian-Somali and Norwegian-Iraqi infants who turned 12 months old during the recruitment period in the three Norwegian counties included. Recruitment challenges are widely reported in studies among immigrant populations [39], and was also observed in the present study. The advantage was the use of bilingual field workers, which enabled the recruitment of mothers with limited Norwegian language skills. A possible limitation is the relatively large number of mothers lost to follow-up at 12 months, although new mothers were recruited. However, a comparison of background characteristics from the 6-month survey [13] and a description of the two immigrant groups by Statistics Norway [40] suggest that the mothers are to some extent representative of Somali-born and Iraqi-born mothers in these three counties in Norway.

In general, the dietary assessment of infants can be complicated by the fact that dietary habits change rapidly in infancy. A review by Burrows et al. has indicated that weighed food records may provide the best dietary estimates for younger children from 0.5 to 4 years old [41]. However, the 24-h multiple-pass recall is less time consuming and is reportedly used more frequently among immigrant populations, along with FFQs [39,42]. The recalls were mainly performed face-to-face, which may be a benefit as potential misunderstandings can be easily clarified in a personal interview. On the other hand, the presence of a field worker may cause a perception of authority on the part of the mothers, which could lead to social desirability response bias [43].

Although the macronutrient composition may reflect the true dietary practices of the two immigrant groups, reporting errors such as underreporting or overreporting may contribute to apparently low or high intakes of certain foods [41]. For infants in particular, not all foods served are necessarily consumed as some is wasted. Furthermore, the mothers may share the responsibility for their children with other adults (e.g., partners, grandparents and day-care), and it may be more difficult for the mothers to assess children’s intake during the hours they were not present [44].

In addition, biochemical indicators of iron and vitamin D status were not measured in the present study, which is necessary in order to assess the infants’ iron and vitamin D status.

## 5. Conclusions

The findings of this study indicate that the food and nutrient intake of Norwegian-Somali and Norwegian-Iraqi infants in general seems to be in accordance with the Norwegian dietary recommendations. The intake of dairy products was generally higher among Norwegian-Somali infants compared to Norwegian-Iraqi infants, but dairy product intake did not seem to affect iron intake. Iron-enriched products and vitamin D supplements were important sources of the infants’ iron and vitamin D intake. Vitamin D supplements and foods rich in iron should continue to be promoted. Infants with immigrant backgrounds are excluded from the Norwegian national dietary surveys, and the present study contributes with information about food and nutrient intake among two of these groups living in Norway. More research to investigate infant feeding practices among immigrant groups in Norway is needed.

## Figures and Tables

**Table 1:** Characteristics of the infants and parents sampled (*n* = 166).

Characteristics	Total (*n* = 166) ^†^		Somali Origin (*n* = 89) ^†^		Iraqi Origin (*n =* 77) ^†^		*p*-Value *
Infants							
Boys/girls ^‡^	55/45		60/40		49/51		0.154
Weight at 12 months (g) ^‡^	10,000	(9286–11,100)	10,270	(9779–11,811)	9708	(9093–10,575)	0.001
Boys	10,120	(9470–11,293)	10,350	(9824–11,800)	9940	(8600–10,885)	0.034
Girls	9815	(9175–10,960)	10,185	(9481–12,104)	9460	(9100–10,500)	0.012
Length at 12 months (cm) ^‡^	77.5	(75.0–79.9)	78.9	(77.0–81.5)	76.5	(74.4–78.3)	<0.001
Boys	78.0	(75.6–80.5)	78.3	(76.8–81.0)	77.8	(74.4–79.3)	0.110
Girls	77.0	(75.0–79.0)	79.5	(76.5–82.8)	76.0	(74.1–77.4)	<0.001
Z-score							
Weight-for-age	0.5	(0.0–1.5)	0.9	(0.2–1.9)	0.4	(0.0–1.1)	0.020
Height-for-age	0.8	(0.8–1.7)	1.4	(0.4–2.4)	0.6	(0.0–1.1)	0.001
Weight-for-height	0.3	(−0.4–1.2)	0.7	(−0.4–1.5)	0.2	(−0.4–0.9)	0.087
Mothers							
Age (years) ^‡^	30.0	(27.0–34.0)	29.0	(27.0–33.0)	32.0	(27.0–36.5)	0.026
Age when immigrated to Norway ^‡^	22.0	(15.0–26.3)	20.0	(12.0–24.0)	23.0	(19.0–29.0)	0.001
Number of years lived in Norway ^‡^	10.0	(5.0–14.0)	10.0	(5.0–15.0)	7.0	(3.0–13.0)	0.012
Education ^‡^							
No/basic education	50		62		36		0.001
High school/higher education	50		38		64		
Current employment status ^‡^							
Not working	79		73		87		0.026
Working (full-time/part-time)	21		27		13		
Primiparous ^‡^	28		28		27		0.891
Fathers							
Origin ^‡^							
Somalia/Iraq	92		95		88		0.102
Other	8		5		12		
Education ^‡^							
No/basic education	28		29		27		0.285
High school/higher education	63		59		68		
Do not know	9		12		5		
Responsible for child during daytime ^‡^							0.021
Mother	58		48		68		
Others (e.g., partner, day-care, etc.)	19		21		17		
Mother and others	23		31		15		

* Comparison of parents and infants of Somali and Iraqi origin. ^†^ Percentages for categorical variables, and medians (25th and 75th percentiles) for continuous variables; ^‡^ Data on gender are missing for three infants from Somalia. The lengths of 44 infants are missing (37 from Somalia and 7 from Iraq). The weights of 42 infants are missing (33 from Somalia and 9 from Iraq). Among male infants, data on length are missing for 26 infants and data on weight are missing for 26 infants. Among female infants, data on length are missing for 16 infants and data on weight are missing for 15 infants. The ages of four Somali-born mothers are missing. The ages when immigrated to Norway are missing for six Somali-born mothers and two Iraqi-born mothers. Mothers’ and fathers’ highest completed education is missing for four mothers and five fathers from Somalia. Current work status is missing for one Somali-born mother and one Iraqi-born mother. Primiparous data and father’s origin is missing for four Somali-born mothers. Who is responsible for the child during daytime is missing for one Norwegian-Iraqi infant. These are not included in the analysis.

**Table 2:** Median intake of foods and drinks (g/day) among all infants at 12 months of age.

	Total (*n* = 166)		Somali Origin (*n* = 89)		Iraqi Origin (*n* = 77)		*p*-Value *
	Median	(P_25_, P_75_)	Median	(P_25_, P_75_)	Median	(P_25_, P_75_)	
Infant foods							
Breastmilk	0	(0, 372)	0	(0, 310)	0	(0, 454)	0.139
Infant formula	0	(0, 190)	0	(0, 103)	0	(0, 275)	0.092
Commercial infant cereal ^†^	125	(0, 275)	200	(102, 300)	58	(0, 150)	<0.001
Commercial infant dinners	0	(0, 0)	0	(0, 0)	0	(0, 0)	0.762
Commercial fruit puree	0	(0, 3)	0	(0, 45)	0	(0, 0)	0.004
Commercial baby drinks	0	(0, 0)	0	(0, 0)	0	(0, 0)	0.884
Bread	17	(4, 31)	18	(0, 31)	16	(6, 31)	0.281
100% sifted flour	0	(0, 0)	0	(0, 0)	0	(0, 4)	0.001
<50% wholemeal flour	0	(0, 0)	0	(0, 0)	0	(0, 8)	<0.001
>50% wholemeal flour	4	(0, 21)	9	(0, 30)	0	(0, 16)	0.055
Grain products	13	(4, 30)	11	(2, 30)	14	(6, 29)	0.268
Cake	0	(0, 7)	0	(0, 0)	6	(0, 15)	<0.001
Potatoes	13	(0, 26)	16	(6, 26)	10	(0, 27)	0.090
Vegetables	37	(22, 66)	41	(26, 67)	33	(16, 64)	0.239
Meat/meat products	17	(0, 35)	5	(0, 23)	24	(11, 49)	<0.001
Fish/fish products	0	(0, 17)	9	(0, 21)	0	(0, 0)	<0.001
Eggs	0	(0, 0)	0	(0, 0)	0	(0, 22)	<0.001
Fruit and berries	43	(7, 90)	36	(0, 58)	58	(22, 121)	<0.001
Yoghurt	41	(0, 94)	57	(0, 110)	25	(0, 63)	0.009
Cheese	2	(0, 12)	1	(0, 11)	3	(0, 13)	0.512
Margarine, butter and oils	4	(2, 8)	4	(2, 8)	4	(2, 8)	0.602
Added sugar	0	(0, 0)	0	(0, 0)	0	(0, 2)	0.045
Drinks							
Cow’s milk	60	(0, 320)	188	(0, 344)	26	(0, 245)	0.025
Whole milk	0	(0, 0)	0	(0, 0)	0	(0, 0)	0.623
Partly skimmed milk	31	(0, 250)	110	(0, 313)	3	(0, 185)	0.013
Skimmed milk	0	(0, 0)	0	(0, 0)	0	(0, 0)	0.127
Nido	0	(0, 0)	0	(0, 0)	0	(0, 0)	0.127
Juice	0	(0, 30)	0	(0, 30)	0	(0, 30)	0.187
Soda/squash with sugar	0	(0, 0)	0	(0, 0)	0	(0, 0)	0.872
Soda/squash, light	0	(0, 0)	0	(0, 0)	0	(0, 0)	0.397
Tea	0	(0, 0)	0	(0, 0)	0	(0, 0)	0.028
Water	180	(120, 270)	160	(98, 248)	187	(126, 299)	0.041

* Comparison of the intake of foods and drinks among infants of Somali and Iraqi origin, Mann–Whitney U test; ^†^ Prepared with water, infant formula, Nido and/or cow’s milk.

**Table 3:** Percentage of consumers and median intake of foods and drinks (g/day) among consumers at 12 months of age.

	Total (*n* = 166)			Somali Origin (*n* = 89)			Iraqi Origin (*n* = 77)			*p*-Value *
	% Consumers	Median	(P_25_–P_75_)	% Consumers	Median	(P_25_–P_75_)	% Consumers	Median	(P_25_–P_75_)	
Infant foods										
Breastmilk	43	434	(310–497)	40	372	(248–455)	47	474	(326–497)	0.414
Infant formula	31	330	(217–540)	26	291	(192–492)	36	385	(240–649)	0.143
Commercial infant cereal ^†^	74	200	(109–300)	88	214	(125–305)	57	140	(75–298)	<0.001
Commercial infant dinners	13	95	(44–148)	14	95	(42–134)	12	98	(45–173)	0.729
Commercial fruit puree	25	50	(45–98)	34	50	(45–100)	14	45	(45–90)	0.004
Commercial baby drinks	2	40	(26–88)	2	40	(–)	3	63	(–)	0.883
Bread	79	23	(11–35)	70	25	(16–36)	90	17	(8–33)	0.002
100% sifted flour	18	12	(5–17)	8	25	(13–38)	30	10	(4–15)	<0.001
<50% wholemeal flour	24	16	(6–23)	12	19	(8–23)	36	13	(5–25)	<0.001
>50% wholemeal flour	52	21	(11–33)	57	25	(15–33)	46	17	(8–33)	0.128
Grain products	84	18	(9–32)	75	18	(9–39)	91	17	(8–31)	0.020
Cake	37	17	(6–19)	16	11	(5–14)	62	14	(6–19)	<0.001
Potatoes	71	20	(12–34)	82	18	(11–28)	58	22	(13–37)	0.001
Vegetables	95	38	(25–68)	94	43	(27–69)	96	34	(18–67)	0.605
Meat/meat products	72	24	(13–46)	60	21	(8–31)	86	32	(16–51)	<0.001
Fish/fish products	46	18	(11–37)	65	16	(10–31)	23	29	(16–43)	<0.001
Eggs	21	28	(13–45)	11	10	(4–34)	33	28	(22–45)	0.001
Fruit and berries	80	55	(31–101)	64	54	(38–88)	99	59	(25–123)	<0.001
Yoghurt	70	63	(36–107)	70	95	(50–112)	70	46	(20–75)	0.948
Cheese	53	10	(4–20)	52	10	(4–18)	55	11	(4–21)	0.713
Margarine, butter and oils	95	4	(2–8)	94	4	(2–8)	96	5	(2–8)	0.605
Added sugar	22	4	(1–8)	17	2	(1–9)	29	4	(2–7)	0.070
Drinks										
Cow’s milk	68	248	(60–420)	71	286	(152–440)	64	121	(29–404)	0.327
Whole milk	17	173	(23–250)	15	245	(179–392)	20	28	(15–170)	0.403
Partly skimmed milk	58	215	(60–368)	65	239	(117–365)	51	180	(32–375)	0.058
Skimmed milk	1	11	(-)	0	-	(-)	3	11	(-)	0.126
Nido	3	156	(68–537)	1	156	(-)	5	169	(49–689)	0.126
Juice	45	31	(19–93)	38	53	(29–105)	52	30	(13–91)	0.076
Soda/squash with sugar	16	31	(15–100)	16	45	(16–100)	17	31	(14–105)	0.841
Soda/squash, light	2	43	(31–58)	3	35	(-)	1	60	(-)	0.385
Tea	8	15	(5–30)	3	63	(-)	13	13	(4–23)	0.021
Water	100	180	(120–270)	100	160	(98–248)	100	187	(126–299)	-

* Comparison of the percentage of consumers among infants of Somali and Iraqi origin, Chi-square test; ^†^ Prepared with water, infant formula, Nido and/or cow’s milk.

**Table 4:** Average energy and nutrient intake among infants of Somali and Iraqi origin at 12 months of age.

	RI ^†^ (1–2 Years)	Total (*n* = 166)		Somali Origin (*n* = 89)		Iraqi Origin (*n* = 77)		*p*-Value *
		Median	(P_25_–P_75_)	Median	(P_25_–P_75_)	Median	(P_25_–P_75_)	
Energy intake (kJ)		3945	(3235–4562)	3791	(2989–4544)	4122	(3454–4751)	0.059
Protein (E%)	10–15	14.5	(12.3–17.0)	14.8	(12.8–17.6)	13.8	(11.7–16.2)	0.074
Total fat (E%)	30–40	35.0	(30.0–39.3)	33.8	(29.9–37.6)	36.5	(32.5–40.9)	0.004
Saturated fat	<10	12.1	(10.1–14.7)	11.7	(9.7–13.7)	12.7	(11.1–15.6)	0.001
Monounsaturated fatty acids		12.6	(10.3–14.5)	11.8	(9.4–13.9)	13.4	(11.3–15.0)	0.002
Polyunsaturated fatty acids		5.7	(4.8–6.8)	5.3	(4.4–6.5)	6.1	(5.3–7.3)	0.003
Total carbohydrates (E%)	45–60	48.5	(44.8–51.9)	49.5	(46.8–51.9)	47.2	(42.9–52.1)	0.048
Added sugars	<10	3.2	(1.3–5.9)	4.0	(1.5–6.1)	2.5	(1.3–5.0)	0.063
Fibre (g)		7.4	(5.4–10.3)	7.4	(5.3–10.2)	7.4	(5.5–10.9)	0.753
Vitamin A (μg)	300 (RAE)	666	(483–878)	717	(574–933)	561	(400–874)	0.011
Vitamin D (μg)	10	12.6	(8.6–17.4)	13.9	(10.2–17.7)	11.7	(7.2–17.0)	0.029
Vitamin E (mg)	4 (μ-TE) ^‡^	10.5	(5.3–14.6)	12.1	(8.5–15.4)	7.3	(4.6–12.3)	<0.001
Thiamine (mg)	0.5	0.61	(0.42–0.80)	0.62	(0.42–0.80)	0.60	(0.41–0.80)	0.864
Riboflavin (mg)	0.6	0.72	(0.44–1.00)	0.77	(0.48–1.08)	0.66	(0.44–0.86)	0.039
Niacin (mg)	7 (NE) ^‡^	6.3	(4.4–8.6)	6.1	(4.5–8.3)	6.5	(4.4–8.7)	0.593
B6 (mg)	0.5	0.56	(0.39–0.75)	0.50	(0.37–0.73)	0.60	(0.44–0.82)	0.033
Folate (μg)	60	95	(72–135)	85	(66–121)	108	(80–151)	0.002
B12 (μg)	0.6	1.8	(0.8–2.9)	2.1	(1.1–3.0)	1.3	(0.6–2.6)	0.022
Vitamin C (mg)	25	72	(46–113)	74	(45–110)	69	(48–121)	0.755
Calcium (mg)	600	727	(519–954)	777	(587–984)	633	(480–938)	0.017
Iron (mg)	8	7.1	(4.6–10.3)	7.3	(4.9–10.3)	6.7	(4.5–11.4)	0.523
Zinc (mg)	5	5.1	(3.9–6.4)	4.8	(3.9–6.0)	5.3	(4.0–6.9)	0.117

* Comparison of infants of Somali and Iraqi origin, Mann–Whitney U test; ^†^ RI = Recommended intake ranges for macronutrients and recommended daily intakes for micronutrients. ^‡^ TE = tocopherol equivalent, NE = niacin equivalent.

**Table 5:** Percentage of Norwegian-Somali and Norwegian-Iraqi infants receiving dairy products and iron-enriched products and median iron intake (mg/day) among infants receiving dairy products and iron-enriched products.

	Total	Somali Origin	Iraqi Origin	*p*-Value ^†^	Iron mg/Day		*p*-Value ^‡^
	(*n* = 166)	(*n* = 89)	(*n*= 77)		Median	(P_25_–P_75_)	
Dairy products							
No (*n* = 21)	13	9	16	0.199	9.4	(5.2–11.1)	0.327
Yes (*n* = 145)	87	91	84		7.0	(4.5–10.2)	
Dairy products							
≤500 g/day (*n* = 140)	84	80	90	0.082	7.2	(4.8–10.7)	0.170
>500 g/day (*n* = 26)	16	20	10		6.4	(4.2–8.9)	
Iron–enriched products *							
No (*n* = 32)	19	12	27	0.015	3.7	(2.6–5.5)	<0.001
Yes (*n* = 145)	81	88	73		8.1	(5.7–11.2)	

* Include infant formula and infant cereals; ^†^ Tested by Chi-square test; ^‡^ Tested by Mann–Whitney U test.
